# Analysis of EMG Signals in Aggressive and Normal Activities by Using Higher-Order Spectra

**DOI:** 10.1100/2012/478952

**Published:** 2012-10-24

**Authors:** Necmettin Sezgin

**Affiliations:** Department of Electrical and Electronics Engineering, Faculty of Architecture and Engineering, Batman University, 72060 Batman, Turkey

## Abstract

The analysis and classification of electromyography (EMG) signals are very important in order to detect some symptoms of diseases, prosthetic arm/leg control, and so on. In this study, an EMG signal was analyzed using bispectrum, which belongs to a family of higher-order spectra. An EMG signal is the electrical potential difference of muscle cells. The EMG signals used in the present study are aggressive or normal actions. The EMG dataset was obtained from the machine learning repository. First, the aggressive and normal EMG activities were analyzed using bispectrum and the quadratic phase coupling of each EMG episode was determined. Next, the features of the analyzed EMG signals were fed into learning machines to separate the aggressive and normal actions. The best classification result was 99.75%, which is sufficient to significantly classify the aggressive and normal actions.

## 1. Introduction

Electromyography (EMG) is the electrical activity of muscle cells and has been used for the classification of actions [1–7], disease detection [8], prosthetic hand control [9], and emotion detection [10]. In this study, 8 channels recorded the EMG signals of 10 aggressive and 10 normal actions of 3 males and 1 female, which were then analyzed for classifying normal and aggressive actions. With this purpose in mind, a composed model of higher-order spectra (HOS) and the learning machine algorithm was proposed. In signal processing, 2nd-order statistics methods such as the power spectrum have gained significant importance. However, many signals have nonlinearity and non-Gaussian behavior, and such signals cannot be examined properly by 2nd-order statistical methods. Thus, higher-order statistical methods have been proved. The HOS was first applied to real signal processing problems in the 1970s and since then it has continued to be applied in many different areas, such as economics, speech signal processing, noisy and artifact removal, biomedical signal processing, and optics. Since EMG signals are nonstationary and non-Gaussian signals, they should be examined by HOS methods. Bispectrum, which is the Fourier transform of the 3rd-order cumulant, can be applied to nonlinear and non-Gaussian signals to extract nonlinear information.

Bispectrum analysis reveals phase information called quadratic phase coupling (QPC). In the present study, the EMG signals analyzed using bispectrum and the QPCs were determined for all of the datasets, and then these QPCs were fed into the extreme learning machine (ELM) algorithm. The ELM is capable of training and testing data fast and with a high accuracy. The main advantage of ELM over the traditional learning methods is that it is very fast due to its algorithm. In the ELM algorithm, the weights between the input layer and the hidden layer and the hidden layer's biases are selected randomly, while the weights between the hidden layer and the output layer are determined analytically. Therefore, considerable time saving is attained in the training stage. Moreover, the performance of the classification method (ELM) was compared with some other machine learning methods, such as support vector machine (SVM), logistic regression (LR), linear discriminant analysis (LDA), and artificial neural network (ANN). The proposed method is satisfactory due to the compared classification methods.

## 2. Material and Methods

### 2.1. Database

In this study, the dataset of the “EMG physical action data set” from the machine learning repository (UCI) [11] was used. 3 male and 1 female subjects took part in the experiment (aged 25 to 30 years), who have experienced aggression in scenarios such as physical fighting. Each subject had to perform 10 normal and 10 aggressive activities. The normal activities were bowing, clapping, handshaking, hugging, jumping, running, seating, standing, walking, and waving, while the aggressive activities were elbowing, front kicking, hammering, headering, kneeing, pulling, punching, pushing, side kicking, and slapping. There were 8 electrodes used, which corresponds to 8 input time series, one for each muscle channel (ch1–8): right bicep (ch1), right tricep (ch2), left bicep (ch3), left tricep (ch4), right thigh (ch5), right hamstring (ch6), left thigh (ch7), and left hamstring (ch8). Each time series contained about 10,000 samples, which were 10 s in length.

### 2.2. Bispectrum Analysis

Bispectrum analysis reveals the phase relation between components of a signal [12–14]. Unlike the power spectrum, the bispectrum is capable of extracting extra information from biological signals such as an EMG signal, which is non-Gaussian and nonlinear. The bispectrum is defined as the Fourier transform of the 3rd-order cumulant.

 The 3rd-order cumulant of a discrete signal *x*(*k*), which is stationary and has a 0 mean, is defined as [14]

(1) C3x(n1,n2)=cum{x(k)x(k+n1)x(k+n2)}=〈x(k)x(k+n1)x(k+n2)〉−〈x(k)〉{〈x(k)x(k+n1)〉+〈x(k)x(k+n2)〉      +〈x(k+n1)x(k+n2)〉}+2〈x(k)〉3,

where 〈·〉 denotes the expected process. 

The *r*th degree moment of *x*(*k*) is defined as

(2) $$\begin{array}{c} m_{rx}\left(n_{1},n_{2},\ldots n_{r-1}\right)=\langle x\left(k\right)x\left(k+n_{1}\right)\cdots x\left(k+n_{r-1}\right)\rangle . \end{array}$$

Thus, (1) can be rewritten as:

(3) C3x(n1,n2)=m3x(n1,n2)−(mx(m2x(n1)+m2x(n2)+m2x(n2−n1))−2mx3).

Alternatively, the 3rd-order cumulant can be written as

(4) $$\begin{array}{c} C_{3x}\left(n_{1},n_{2}\right)=m_{3x}\left(n_{1},n_{2}\right)-m_{3x}^{G}\left(n_{1},n_{2}\right), \end{array}$$

where *m*_3*x*_(*n*_1_, *n*_2_) is the 3rd-order moment function of *x*(*k*) and *m*_3*x*_^*G*^(*n*_1_, *n*_2_) is the 3rd-order moment function of a Gaussian random process with the same 1st- and 2nd-order characteristics of *x*(*k*)

(5) m3xG(n1,n2) =mx(m2x(n1)+m2x(n2)+m2x(n2−n1))−2mx3.

An important result of (4) is that if *x*(*k*) is a Gaussian process, then its 3rd-order cumulant is 0 [14, 15]:

(6) $$\begin{array}{c} m_{3x}\left(n_{1},n_{2}\right)=m_{3x}^{G}\left(n_{1},n_{2}\right),\\ \text{then}\;C_{3x}\left(n_{1},n_{2}\right)=0. \end{array}$$

The 3rd-order cumulant and 3rd-order moment of a process, which has a 0 mean, are equal to each other. Thus, (4) becomes

(7) $$\begin{array}{c} C_{3x}\left(n_{1},n_{2}\right)=m_{3x}\left(n_{1},n_{2}\right). \end{array}$$

The correlation is a relation between 2 points, whereas the 3rd-order cumulant is a relation between combinations of 3 points in a time series. The 3rd-order cumulant has symmetry properties as

(8) C3x(n1,n2)=C3x(n2,n1)=C3x(−n1,n2−n1)=C3x(n1−n2,−n2).

The Fourier transform of the 3rd-order cumulant is bispectrum and defined as

(9) $$\begin{array}{c} B\left(\omega_{1},\omega_{2}\right)=\sum_{n_{1}=-\infty }^{\infty }\;\sum_{n_{2}=-\infty }^{\infty }C_{3x}\left(n_{1},n_{2}\right)W\left(n_{1},n_{2}\right)e^{-j(\omega_{1}n_{1}+\omega_{2}n_{2})},\\ \left|\omega_{1}\right|,\left|\omega_{2}\right|\leq \pi , \end{array}$$

where *W*(*n*_1_, *n*_2_) is the 2-dimensional window function that decreases the variance of the bispectrum. In this study, a Hanning window, which is 0.05 s in duration, was used. Equation ([Disp-formula EEq9]) can also be defined in the Fourier transform of *x*(*k*) as

(10) $$\begin{array}{c} B\left(\omega_{1},\omega_{2}\right)=\langle X\left(\omega_{1}\right)X\left(\omega_{2}\right)X^{*}\left(\omega_{1}+\omega_{2}\right)\rangle ,\; \end{array}$$

where * denotes a complex conjugate. 

*B*(*ω*_1_, *ω*_2_) is a symmetric function, such that a triangular region 0 ≤ *ω*_2_ ≤ *ω*_1_, *ω*_1_ + *ω*_2_ ≤ *π* could completely describe the whole bispectrum. The other regions in the bispectrum are the symmetry of the defined triangular region. A peak observed in the triangular region indicates that the energy component at frequency (*ω*_1_, *ω*_2_) is produced, likely due to the quadratic nonlinearity dependence, called QPC [16]. On the contrary, a flat bispectrum at the 2 frequency components *ω*_1_ and *ω*_2_ suggests no such activities. Consequently, phase coupled components contribute extensively to the 3rd-order cumulant sequence of a process. This unique capability of bispectral analysis becomes a useful tool to detect and quantify the possible existence of QPCs in the EMG signals of aggressive activities. To quantify the QPC, one can take advantage of the quantification of non-Gaussianity, which has a direct relation to phase coupling, of a random process as the sum of the magnitudes of the estimated bispectrum given by [17]:

(11) $$\begin{array}{c} D=\sum_{\left(\omega_{1},\omega_{2}\right)}\left|B\left(\omega_{1},\omega_{2}\right)\right|;\;\omega_{1}\neq \omega_{2}. \end{array}$$

The bispectrum quantity of all of the episodes in the database was determined through (11) and fed as input into the ELM classifier in order to separate aggressive activities from normal activities.

### 2.3. Extreme Learning Machine Algorithm

In the ELM, the network has 3 layers: input, output, and 1 hidden layer. The weights between the input and hidden layers *W*_*i*_ = (*W*_*i*1_, *W*_*i*2_,…, *W*_*in*_) and the hidden layer biases *b*_*i*_ are selected randomly and *H*, the hidden layer output matrix, is determined analytically. In the ELM, the training of the network is to minimize the sum square error for the *Hβ* = *Y* as

(12) ||H(W1,…,WM;b1,…,bM)β∧−Y|| =βmin⁡||H(W1,…,WM;b1,…,bM)β−Y||,

where $\hat{\beta }=H^{+}Y$ is the minimum square form of *Hβ* = *Y* and *β* is the weights between the hidden and output layers. *H* ^+^ is the inverse of the generalization Moore-Penrose of *H*. The $\hat{\beta }$ estimations are the minimum of the solution of the sum square of *Hβ* = *Y*.

The ELM does not only find the minimum error, but can also achieve the best performance with respect to conventional gradient based methods. This performance arises by the singularity of the matrix *H* ^+^. $\hat{\beta }$ is a singular solution. The ELM algorithm can be summarized in 3 steps as follows [18].

- The weights *W*_*i*_ = (*W*_*i*1_, *W*_*i*2_ … *W*_*in*_), which are between the input layer and the hidden layer, and the hidden layer biases *b*_*i*_, are selected randomly.
- The output of the hidden layer, *H*, is determined.
- The weights $\hat{\beta }$, which are between the hidden layer and the output layer, are calculated as $\hat{\beta }=H^{+}Y$, where *Y* is the target vector.

## 3. Results

In the present study, the ELM was used in order to classify the EMG signals as either belonging to an aggressive action or a normal action. In the 1st stage of the QPC, each 10 s EMG episode was determined by bispectral analysis. After bispectral analysis of the EMG signal, in the 2nd stage, the extracted features, which are the QPC quantity, were fed into the input of the ELM classifier. For the ELM algorithm, the training-testing rate was randomly chosen as 50%-50% from the extracted features of the EMG. 

**Figure 1:**
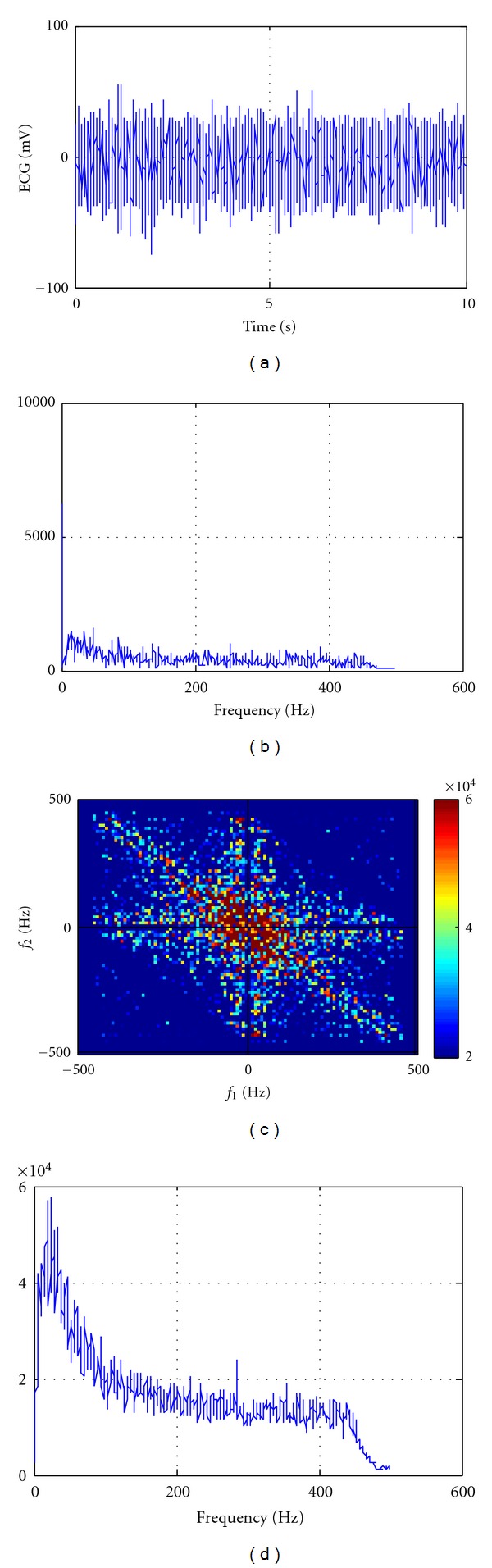
(a) The EMG activity of normal action, (b) its power spectrum, (c) its bispectrum, and (d) its bispectrum in 2 dimensions.

**Figure 2:**
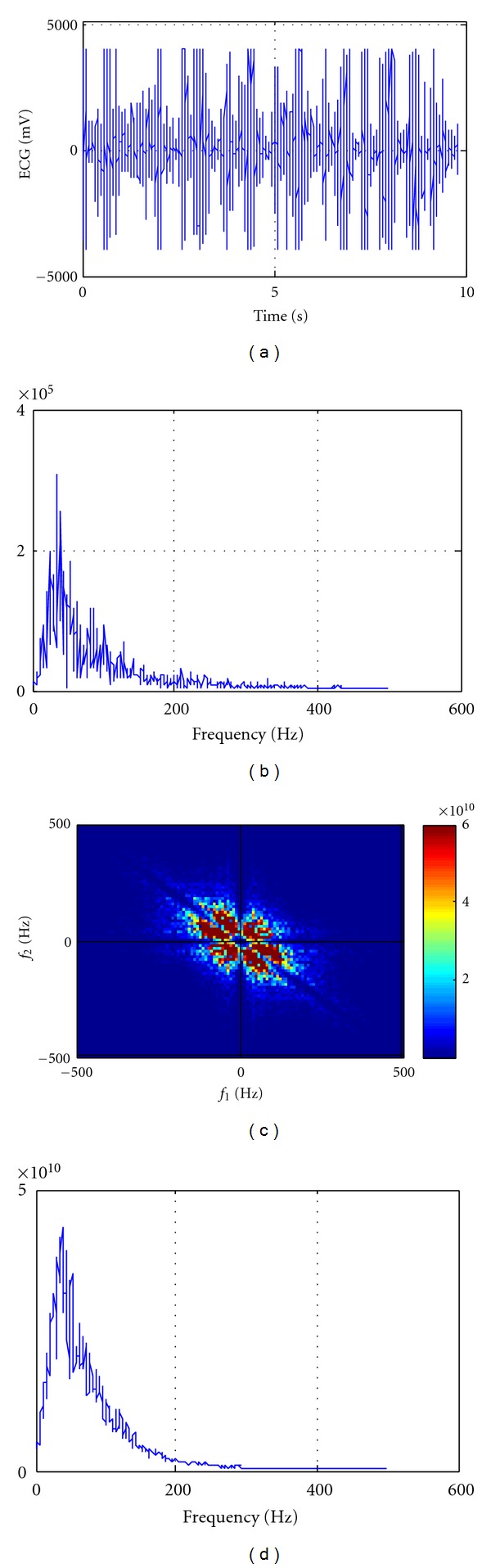
(a) The EMG activity of aggressive action, (b) its power spectrum, (c) its bispectrum, and (d) its bispectrum in 2 dimensions.

 An example of normal EMG activity (waving) and aggressive activity (frontkicking) is shown in Figures 1 and 2, respectively. In Figures 1 and 2, normal and aggressive EMG actions (Figures 1(a) and 2(a)) and their corresponding power spectrums (Figures 1(b) and 2(b)), bispectrums (Figures 1(c) and 2(c)), and bispectrums in 2 dimensions (Figures 1(d) and 2(d)) are shown, respectively. 

 As shown in Figure 1, the bispectrum (Figure 1(d)) is about 20 times higher than the power spectrum (Figure 1(b)), and in Figure 2, the bispectrum (Figure 2(d)) is about 100,000 times higher than the power spectrum (Figure 2(b)). This means that the nonlinearity and non-Gaussian signals are increased rapidly by aggressive actions. Accordingly, the bispectrum of aggressive activity (Figures 2(c) and 2(d)) is much higher than normal activity (Figures 1(c) and 1(d)). Furthermore, the nonlinearity and non-Gaussian concentration of aggressive activity (Figure 2(c)) are centered at lower frequencies than normal activities (Figure 1(c)).

The ELM algorithm built in this study has 1 node in the input layer, 40 nodes in the hidden layer, and 2 nodes in the output layer. The activation function was sigmoid since it allowed the best performance. The number of nodes in the hidden layer and the type of activation function were chosen by trial and error. The classification accuracy for the ELM was 99.75% and the duration of the training-testing phases was 0.07 s and 0.005 s, respectively. The same data were also classified by ANN, SVM, LR, and LDA classifiers. Their results and a comparison with the ELM is shown in Table 1. It is clear that the ELM is faster and has higher accuracy performance than conventional learning machines.

## 4. Discussion and Conclusions

In the present study, aggressive and normal EMG signals were analyzed using bispectrum and the EMG signals were classified in aggressive and normal activities using learning machine algorithms. A best performance was obtained using the ELM algorithm, which has an accuracy of 99.75%. The bispectrum of the aggressive and normal activities of the EMG were analyzed and the QPC quantities of each episode were determined and fed into the input of the learning machines. Aside from the classification of the aggressive and normal activities, a comparison of the power spectrum and bispectrum was performed in the EMG signals. Thus, the bispectrum of the EMG signal is a candidate to separate aggressive and normal activities. The results obtained from the ELM classifier are acceptably high enough to differentiate aggressive activities from normal activities. This simple and effective method may help experts in defining aggressive activities and this can give important clues about some abnormalities related to EMG signals.

## Figures and Tables

**Table 1:** Performances of the ANN, SVM, LR, LDA, and ELM learning machines.

Model	Training process time (s)	Testing process time (s)	Accuracy (%)
ANN	32.25	1.18	98.20
SVM	1.80	0.20	96.15
LR	0.10	0.05	97.50
LDA	0.09	0.04	97.25
**ELM**	**0.07**	**0.005**	**99.75**
